# Sequestration of ubiquitous dietary derived pigments enables mitochondrial light sensing

**DOI:** 10.1038/srep34320

**Published:** 2016-10-12

**Authors:** Dan Zhang, Kiera Robinson, Doina M. Mihai, Ilyas Washington

**Affiliations:** 1 Columbia University Medical Center, Ophthalmology, New York, NY 10032, USA

## Abstract

Animals alter their physiological states in response to their environment. We show that the introduction of a chlorophyll metabolite, a light-absorbing pigment widely consumed in human diets, to *Caenorhabditis elegans* results in animals whose fat mass can be modulated by exposure to light, despite the worm consuming the same amount of food. In the presence of the chlorophyll metabolite, exposing the worms to light increased adenosine triphosphate, reduced oxidative damage, and increased median life spans, without an effect on animal reproduction. Mice fed a dietary metabolite of chlorophyll and exposed to light, over several months, showed reductions in systemic inflammation as measured by plasma α-macroglobulin. We propose that dietary chlorophyll metabolites can enable mitochondria to use light as an environmental cue, by absorbing light and transferring the energy to mitochondrial coenzyme Q.

Interactions with environmental factors such as light, gravity, the earth’s magnetic field, food, microbes, and the atmosphere are, for all practical purposes, inescapable. One's genetic makeup, expression patterns of genes, composition of molecules, biochemistry, physiology and morphology can all be viewed as culminations of adaptations to the environment. Evolutionarily conserved environmental cues can be defined as environmental factors that humans have become to rely upon for normal function. If so, then the identification of such cues and the determination of how the frequency of their exposure can affect physiology can be expected to be important towards maintaining human health, wellbeing, and disease prevention. Nutrition and electromagnetic radiation from the sun are conserved environmental cues that affect every living organism. In this work, we asked how these two cues might interact inside the body to perturb physiology.

Respiration, in animal cells, is enabled by transmembrane proteins linked by diffusible coenzyme Q_10_ (CoQ_10_) molecules in the mitochondrial inner membrane space. We proposed that animal mitochondria could sequester certain metabolites of dietary chlorophyll, where once inside, the metabolites could absorb long wavelength light (LWL) and transfer the absorbed energy to CoQ_10_, resulting in the photoreduction of CoQ_10_^1^. In the proposed scenario, dietary metabolites of chlorophyll would play a similar role, in animals, as chlorophyll plays in the chloroplatst of plants: where photon absorption by chlorophyll results in the photoreduction of plastoquinone, the plant equivalent to CoQ_10_. The potential photoreduction of CoQ_10_ mediated by dietary pigments is of interest because mitochondria play a central role in coordinating physiology with environmental demands^2^,^3^,^4^, and the oxidation state of CoQ_10_ provides a known molecular basis for signal origin in cell signaling^5^. Thus, the photoreduction of CoQ_10_ by an accessory pigment would offer a way to potentially modulate physiology in response to environmental light. For example, by sequestration of the chlorophyll metabolite pheophorbide-a (PA), Caenorhabditis elegans are able to absorb LWL, resulting in increased ATP concentrations and median life spans^1^. Here, we investigated additional changes induced by LWL in the presence of PA. We were interested in the extent to which wavelengths of light and dietary pigments, which are both present in the body, but have largely been regarded as benign, might affect the functioning of an organism. This work has implications as to how the frequency of exposure to light and chlorophyll plant pigments may enable normal function.

## Results

### Light and a dietary pigment modulates CoQ_10_ ratios and adenine nucleotides in animal mitochondria

**Figure 1:**
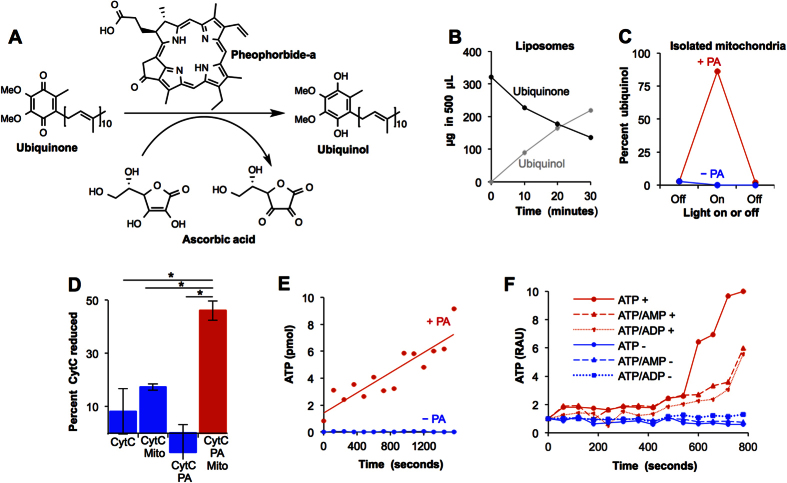
Photoreduction of coenzyme Q (CoQ_10_) activates complex III, which reduces cytochrome c, resulting in generation of ATP, in animal mitochondria. (**A**) Schematic of pheophorbide-a (PA) catalyzed photoreduction of coenzyme Q (also known as ubiquinone/ubiquinol). (**B**) Photoreduction of ubiquinone in liposomes catalyzed by PA. The x-axis represents the amount of time the liposome mixture was exposed to red light. (**C**) Photoreduction of ubiquinone in isolated, intact heart mitochondria. Mitochondria were either treated with PA (+) or not treated with PA (−). When exposed to light, 86% of CoQ_10_ was reduced to ubiquinol in PA treated mitochondria (red line) but not for control mitochondria (blue line). When the light was turned off, ubiquinol was reoxidized. (**D**) Photoreduction of ubiquinone activates complex III resulting in the reduction of cytochrome C. Reaction conditions noted on the x-axis. Values represent the amount of cytochrome c reduced upon exposure to light minus the amount of cytochrome c reduced when the same samples were kept in the dark. Averages of 8 replicates and standard deviations are shown. CytC: cytochrome c; Mito: mitochondria; *P-value < 0.05 determined by unpaired Student’s t-test. (**E**) Storage of photonic energy by animal mitochondria. ATP synthesis in the PA treated (+) and control (−) mitochondria exposed to red light as measured by the luciferase assay. ATP synthesis was coupled to CoQ_10_ photoreduction. (**F**) ATP concentrations and ATP/AMP and ATP/ADP ratios, as measured by UPLC in the light exposed, PA treated heart mitochondria (+, red lines), were consistent with ATP synthesis from ADP and AMP pools. PA-naïve, light exposed mitochondria (−, blue lines) showed no change in adenosine nucleotides.

To show that metabolites of dietary chlorophyll, or PA, could catalyze the photoreduction of CoQ_10_, we incorporated CoQ_10_ into liposomes with less than stoichiometric amount of PA, in the presence of vitamin C, used as a hydrogen donor. Upon exposure of the solution to light centered at λ _max_ = 660 nm to simulate the long wavelength portion of environmental lighting, ubiquinone (the oxidized form of CoQ_10_) was increasingly reduced to ubiquinol (the reduced form of CoQ_10_) with increasing light dose (Fig. 1A,B). In the absence of vitamin C, PA, or light, no reduction occurred. A few hundred times more molecules of ubiquinone were reduced than molecules of added PA, suggesting that PA acted catalytically. Similarly, when PA-treated isolated mitochondria were exposed to LWL, 86% of mitochondrial CoQ_10_ was reduced to ubiquinol, consistent with the results in liposomes (Fig. 1C). Before LWL exposure, nearly all the CoQ_10_ was in its oxidized form. When the light was turned off, the generated ubiquinol was oxidized to ubiquinone. LWL had no effect on the ubiquinone:ubiquinol ratios in the absence of PA.

After demonstrating that photon absorption by PA could photoreduce ubiquinone, we sought to determine the extent to which the generated ubiquinol were in a position to donate electrons to complex III, the next step in electron transport. We measured complex III activity by its ability to reduce cytochrome c in the presence of rotenone, to limit the reduction of CoQ_10_ by complex I, and by potassium cyanide to limit the oxidation of cytochrome c by complex IV. In the presence of PA, within 15 minutes of LWL exposure, 45% of cytochrome c was reduced (Fig. 1D). However, when kept in the dark or in the absence of added PA or mitochondria, significantly less cytochrome c was reduced. This suggested that the photoreduced CoQ_10_ was available to participate in electron transport.

To show that ubiquinone photoreduction and subsequent cytochrome c reduction could be coupled with ATP synthesis, we measured the concentrations of adenosine nucleotides in the same mitochondria. Quantification of ATP by the luciferase assay revealed that ATP concentrations increased with light exposure in PA -treated mitochondria but not in PA naïve mitochondria (Fig. 1E). The number of photons put into 50 μL of the reaction mixture was approximately 52 pmol per second (the light intensity was 4 μmol s^−1^ m^−2^, and 50 μL is 0.000013 m^2^). The total rate of ATP synthesis in 50 μL was 0.0037 pmol per second (total protein in 50 μL was 81 ± 6 μg). Quantification of adenosine nucleotides by Ultra Performance Liquid Chromatography (UPLC) was consistent with that of the firefly luciferase assay (Fig. 1F). As ATP increased, ATP:ADP and ATP:AMP ratios increased, consistent with an increase in ATP from ADP and AMP pools. In contrast, in control PA naïve mitochondria, no changes in ATP, ADP, and AMP were observed upon light exposure, further confirming measurements by the luciferase assay. The concentrations of adenosine nucleotides remained unchanged when the PA treated mitochondria were kept in the dark. The above data suggested that the isolated mitochondria were able to convert approximately 0.01% of incident photons into stored energy as ATP (i.e. 14,000 photons of incident light could produce one molecule of ATP).

### Light and a dietary pigment modulate metabolism in an animal model

**Figure 2:**
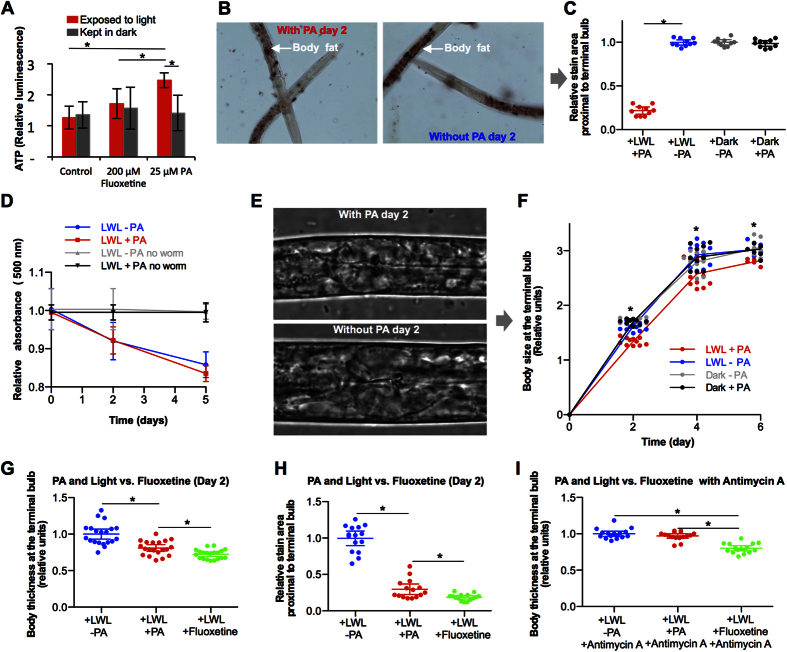
Light and PA regulates leanness and fat mass in *C. elegans.* *P-value < 0.05 determined by unpaired, two sided, Student’s t-test. Replicate measures are shown with averages and 95% confidence intervals. (**A**) ATP quantification in animals kept in the dark or exposed to LWL in the presence or absence of PA and/or the drug fluoxetine. (**B**) Oil Red-O staining of fat mass. From hatching, animals were raised on wild bacteria/fungi and exposed to light centered at λ_max_ = 660 nm, to simulate the long wavelength portion environmental lighting, for 12 hours followed by 12 hours of darkness (cylic light). Representative images taken at 400 x magnification. (**C**) Quantification of Oil Red-O staining at 2 days of age. (**D**) Food consumption in light and PA treated animals compared to control cohorts. (**E**) Representative images of a 2-day old worm raised on wild bacteria/fungi under cyclic light in the presence of PA (lean worm) compared to a PA naïve animal raised under cyclic light. (**F**) Average body thickness measured at terminal bulb. (**G**) Average body thickness measured at terminal bulb. Comparison of leanness triggered by light/PA with that by fluoxetine after 2 days of treatment. (**H**) Quantification of Oil Red-O staining in worms treated with light/PA or fluoxetine for 2-days. (**I**) Average body thickness measured at terminal bulb in the presence of complex III blocker antimycin.

*C. elegans* provide a convenient animal model to study energy homeostasis. A number of pathways central to regulation of mammalian metabolism appear to have similar function in *C. elegans*^6^. In the wild, *C. elegans* coexist with rich microbial communities^7^ which dictate almost every aspect of *C. elegans* biology^8^. We therefore conducted experiments with worms raised on a wild culture of bacteria/fungi (Supplementary Fig. 1A). Upon co-incubation, PA fluorescence was seen by fluorescence microscopy as a diffuse staining throughout the animal (Supplementary Fig. 1B,C) indicating that *C. elegans* readily incorporated PA. Subsequent exposure to LWL increased ATP concentrations by 75% in PA treated animals but not in PA naïve animals or in animals treated with fluoxetine (Fig. 2A), a drug that leads to alterations in *C. elegans fat stores*^9^. This indicated that LWL, in the presence of PA, could alter energy homeostasis in the worm *in vivo*, as well as in isolated mitochondria.

In *C. elegans,* fat is primarily stored in intestinal and epidermal cells. The size of these lipid pools, as determined by staining with lipophilic dyes, has been used as an indicator of the energetic state of the worm. Two-day old PA and light treated worms had 78% less fat mass as quantified by Oil Red O staining (Fig. 2B,C, and Supplementary Fig. 2A). Fat deposits were similar in all the three control groups: PA naïve animals kept under cyclic light, and PA treated and PA naïve animals kept in the dark. This trend continued such that at 4 and 6 days of treatment, the PA and light treated *C. elegans* contained 91% and 84% less fat mass respectively, compared to PA naïve animals exposed to cyclic light (Supplementary Fig. 2B and Table 1). This observation of light and PA exposed animals having less fat mass was consistent, regardless of when the experiments were conducted or changes made to the food source (Supplementary Figs 3 and 4), indicating that light induced reduction in fat mass, in the presence of PA, were independent of the community of wild bacteria/fungi. Fat mass reflects caloric intake, energy expenditure, caloric absorption, and storage. Lean worms consumed the same amount of food as non-lean worms (Fig. 2D), suggesting that reductions in fat mass where not a result of significantly decreased caloric intake.

Because fat mass can also influence body size, we also measured the width of animals proximal to the terminal bulb. Two-day old worms, which had been treated with PA at the L1 stage and exposed to 12 hours of LWL and 12 hours of darkness (cyclic light) for 2 days, were 19% to 24% thinner compared to PA naïve animals kept under the same cyclic light, or to worms kept in the dark in the presence or absence of PA (Fig. 2E,F, and Supplementary Fig. 5 and Table 2). In the three control groups, the worms were the same width, suggesting that both light and PA were necessary to bring about leanness. The PA and light exposed worms were also thinner than the three control cohorts when measured at 4 and 6 days of cyclic light when the experiment ended. Although the PA and light treated animals were thinner, their width increased at the same rates compared to the control worms (Fig. 2F, 0.62 ± 0.04 relative units per day, between 2 and 4 days, for PA and light treated animals vs. 0.65 ± 0.03 for controls, P-value = 0.45). Thus, the treatment did not affect growth rates. The observations that light alone did not significantly effect leanness, suggested that reductions in leanness were not simply through modulation of circadian rhythms^10^.

**Figure 3:**
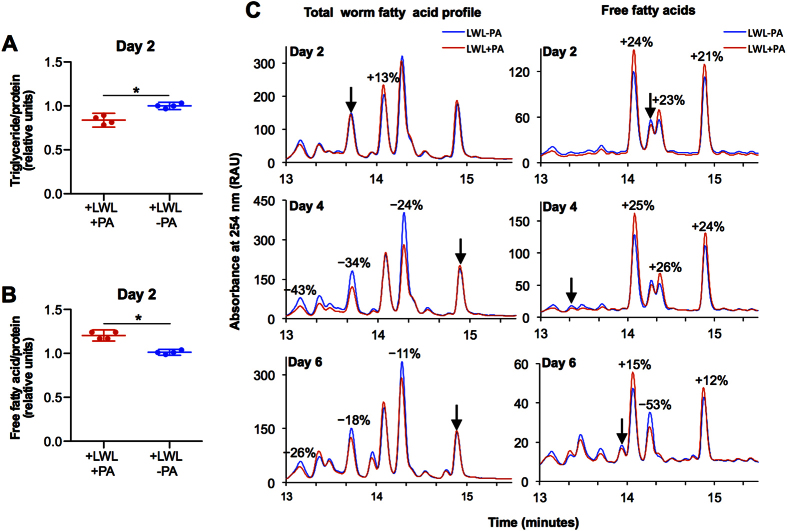
Light and PA regulates lipid composition in *C. elegans.* (**A**) Triglyceride quantification in light exposed animals in the presence or absence of PA at 2 days of age. Replicate measures are shown with averages and 95% confidence intervals. *P-value < 0.05 determined by unpaired, two sided, Student’s t-test. (**B**) Total free fatty acid quantification in worms described above. (**C**) UPLC profiles illustrating the relative amounts of fatty acids in light exposed animals in the presence or absence of PA at 2, 4, and 6 days of age. Free fatty acids represent the free fatty acids in the worm, while the total fatty acid profile represents free fatty acids obtained after saponification of a worm extract. Each peak represents a different free fatty acids. Peaks eluting later in time represent longer fatty acid chain lengths or more hydrophobic lipids. Numbers above the peaks represent the percent change in peak area (i.e. fatty acid amount) between the light exposed PA treated and PA naïve animals. Arrows point to peaks with the same area, to which curves where normalized.

In the presence of PA, light was able to induce leanness to a similar extent compared to fluoxetine as determined by both physical measures of body width (Fig. 2G) and by Oil Red O staining of fat mass (Fig. 2H). Alterations in mitochondrial energetics (CoQ ratios and ATP concentrations) and alterations in worm ATP concentrations upon light stimulation, in the presence of PA, are all consistent with mitochondrial induced signaling. Inhibiting mitochondrial complex III with the drug antimycin A prevented reductions in leanness in the light exposed and PA treated animals, but not in the fluoxetine treated animals (Fig. 2I), further supporting the notion of a primary perturbation in mitochondrial energetics. After establishing that worms exposed to LWL in the presence of PA were thinner and had less fat mass, we investigated the extent to which lipid profile were affected. Enzymatic quantification of total triglycerides (Fig. 3A) agreed with Oil Red O staining. At 2 days of treatment, we observed more free fatty acids in the PA and light treated worms as compared to light treated worms in the absence of PA (Fig. 3B). Quantification of lipid profiles by Ultra Performance Liquid Chromatography (UPLC) revealed up to 16 different major lipids. Light and PA exposed worms had altered ratios of certain lipids at 2, 4, and 6 days of treatment compared to PA naïve animals exposed to cyclic light (Fig. 3C).

**Figure 4:**
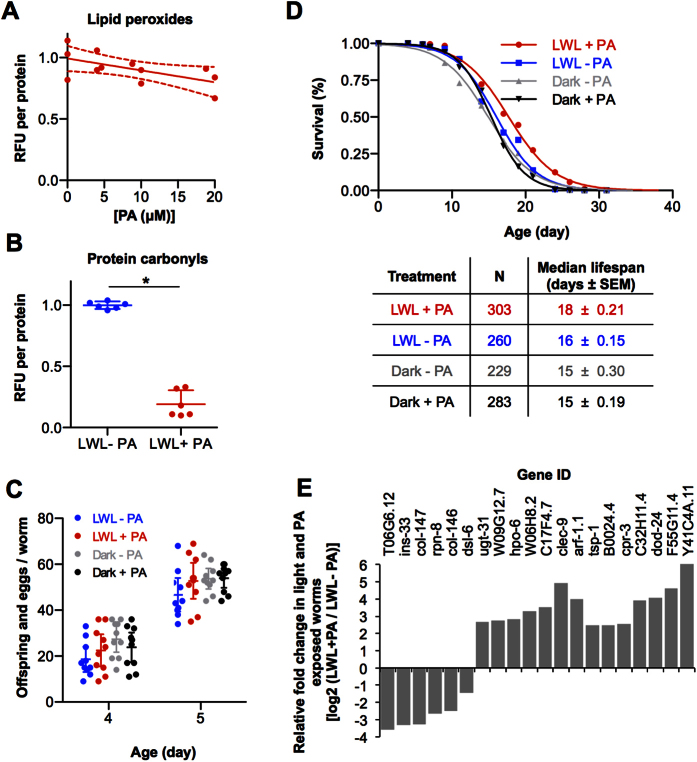
Light and PA did not show overt toxicity. (**A**) Quantification of lipid peroxides in animals raised on wild bacteria/fungi under cyclic light in the presence or absence of PA for 2 days. Replicate measures are shown with averages and 95% confidence intervals. *P-value < 0.05 determined by unpaired, two sided, Student’s t-test. (**B**) Quantification of protein carbonyls in the worms described in panel A. (**C**) Number of offspring (eggs and worms) per adult worm at 4 and 5-days of treatment. (**D**) Medium life spans. (**E**) Fold change in RNA transcripts as determined by RNA-sequencing in light exposed, 2-day old animals in the presence PA relative to light exposed animals in the absence of PA. Significance was defined by a Q-value < 0.05.

Because excitation of PA by light could lead to the formation of singlet oxygen and oxidative damage, we also looked at signatures of oxidative damage. Light exposed, PA treated animals showed less oxidative damage compared to light exposed PA naïve animals as measured by 12% less lipid carbonyls (Fig. 4A) and 80% less protein carbonyls (Fig. 4B, 1 ± 0.01 control vs. 0.19 ± 0.04 treated, P-value = <0.05). The reproductive capacity of the worms was unchanged upon exposure of the worms to PA and light (Fig. 4C). Additionally, we observed 20% longer median lifespans in the PA and light exposed worms compared to light exposed PA naïve animals and animals raised in the dark (Fig. 4D). Taken together, these results suggested that PA and light treatment was not overtly detrimental to the worm and was conversely more consistent with a net positive effect.

### Immune function associated with exposure to PA and light

We interrogated the transcriptomes of the animals raised under cyclic light for 2 days in the presence and absence of PA. We obtained reliable expression for 19,549 genes by sequencing polyadenylated RNA transcripts. Of these, 661 genes were differentially expressed in the light and PA group compared to the control group, using a p-value cutoff of ≤ 0.05. Using the adjusted p-values (Q-value < 0.05)^11^, of the 661 genes, 6 transcripts were significantly down-regulated and 14 were significantly up-regulated (Fig. 4E). The most down-regulated genes were *col-147* and *col-146.* The human ortholog being a lung collectin surfactant protein, SFTPD (surfactant protein D)^12^, which is involved in the innate immune response^13^. This finding suggested lower immune activity in the PA and light treated worms, which was also consistent with the up-regulation of *ugt-31*. These same genes are differentially regulated in the worm, in the opposite directions, during pathogen infection^14^. Further, half of the up-regulated transcripts (7 of the 14) encoded for genes involved in host defense.

**Figure 5:**
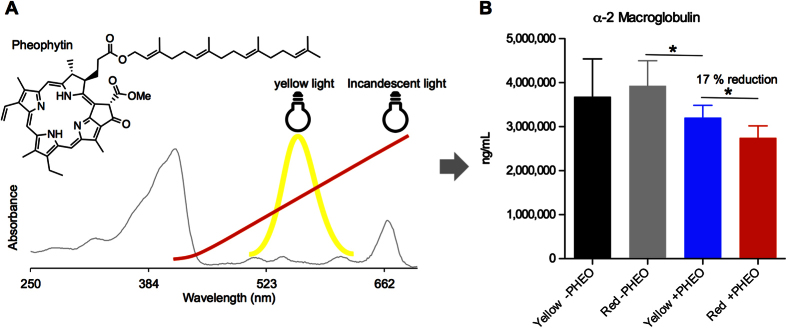
Exposure to light and pheophytin associated with a reduction in inflammation. (**A**) SKH1-Elite, hairless mice, pictured, were fed a chlorophyll free diet. For two groups of mice, we added pheophytin to the diet; the absorption spectrum of which is shown. Animals were then raised either under yellow light, which minimally excites pheophytin, or incandescent light, which contains red wavelengths, that can excite pheophytin. Approximate absorption bands of the lights used are shown. (**B**) Plasma α-2 macroglobulin levels in the above described, 4 groups of mice (n = 5 to 7), after 4 months of treatment. Averages and 95% confidence intervals are shown. *P-value < 0.05, determined by unpaired, two sided, Student’s t-test.

Based on the above analysis, we hypothesized that exposure to PA and light associated with inflammatory status. To test this hypothesis, we administered a diet with and without the chlorophyll derivative pheophytin to immunocompetent, hairless mice. We then raised each group of animals under incandescent light, which is able to excite pheophytin (or any of its major dietary derivatives), or yellow light, which only minimally excites pheophytin, in 12 hour light dark cycles, for four months (Fig. 5A). We fed pheophytin because demetallization of chlorophyll to form pheophytin readily happens in the gut and we have previously shown that animals given a chlorophyll rich deist, accumulate chlorophyll metabolites throughout their bodies^1^. Each ≈ 30 gram animal consumed ≈ 6 mg of pheophytin per day. After 4 months, we interrogated plasma acute phase proteins. Animals exposed to incandescent light and given pheophytin, had 17% lower plasma α-macroglobulin compered to animals given pheophytin but kept in the dark (Fig. 5B). In the absence of pheophytin, yellow or incandescent light had no effect on plasma α-macroglobulin. Pheophytin alone, reduced plasma α-macroglobulin but this reduction was more pronounced in animals exposed to incandescent light. A 17% reduction in α-macroglobulin is similar, in magnitude, to the increase in plasma α-macroglobulin in mice injected with zymosan or LPS, known stimulants of the innate immune system^15^. We did not obverse significant changes in plasma, adipsin, C-reactive protein, haptoglobin, amyloid P, and α-1 acid glycoprotein amongst the 4 groups of animals (Fig. S6).

## Discussion

LWL, of approximately 660 nm, penetrates most biological tissues, relatively unabsorbed. Here we propose a potential mechanism by which this photonic energy might be utilized by the sequestration of dietary pigments, which are able to absorb this energy. The measured 14,000 photons to generate one molecule of ATP was estimated using total incident flux (as opposed to the fraction of light absorbed), and is presumably on the low end taking into account changes in mitochondria architecture during isolation and handling. To gain insight into the extent to which the absorption of this additional energy, by an exogenous chromosphere, might affect an animal, we looked at lipid regulation in *C. elegans* because lipids act as energy reserves. We were able to show that the absorbed photonic energy resulted in changes in the worm’s lipid deposits, the concentrations of total triglycerides and free fatty acids, lipid ratios, and worm thickness. We observed sustained metabolic perturbations in lipids up to 6 days of age. This seems rare for this model where reductions in fat mass are mostly reported up to around 2 to 3 days of age^9^,^16^,^17^,^18^,^19^,^20^,^21^,^22^,^23^. The observed light induced reductions in fat mass^16^,^17^,^18^,^19^,^20^ and alterations of lipid profiles^21^,^22^ were greater or about the same in magnitude as reported for changes in response to genetic engineering or through exogenous control of metabolism. For example, out of a screen of 3,200 chemicals, 5 (0.16%) lowered fat mass more than two-fold relative to control-treated animals^9^. However, in contrast to foreign chemicals, here we are able to show control of metabolism resulting from exposure to environmental cues that have a normal presence inside the body. This opens the possibility that similar chemistry may be taking place in animals and may constitute normal function. Differences in ATP, transcriptomes, a decrease in oxidative damage, and increased in lifespans, all suggest that the additional absorbed energy can impact animal physiology.

In the majority of metabolic studies, *C. elegans* were raised on what can be considered purified diets or in simple environments: using *E. coli* OP50 as a sole food source and controlling or eliminating bacterial and fungal growth. However, animals raised on purified diets are generally more susceptible to external perturbations^24^,^25^ and*C. elegans* are naturally colonizers of nutrient- and bacteria-rich substrates^7^ and their body size, fat stores, lipid composition, behavior, and life-spans are all dependent on the bacteria diet^8^. Here, we observed changes in energetics for *C. elegans* raised in a complex environment rich in bacteria/fungi, suggesting that these invoked changes were not restricted to *C. elegans* cultured on strict artificial diets/environments and the invoked changes were larger than diet induced variability.

Upon excitation by LWL, PA can either lose their excitation energy as heat, or transfer the excitation energy to a neighboring molecule. Transfer of excitation energy to oxygen can result in the formation of singlet oxygen (a reactive oxygen species), which could have potentially played a role in the observed phenotypes through increased oxidative stress. However, measurements of oxidative damage suggested that light, in the presence of PA, did not obviously increase oxidative stress. To the contrary, we observed a decrease in lipid and protein carbonyls in the light and PA treated animals. We also did not observe any obvious deleterious effects of light and PA on ATP levels, feeding, growth, reproduction, or life spans. Taken together, these results suggest that the observed phenotypes were not the result of general sickness, altered feeding behavior, or oxidative damage, although the possibility of stress response mechanisms cannot be ruled out at this time.

**Figure 6:**
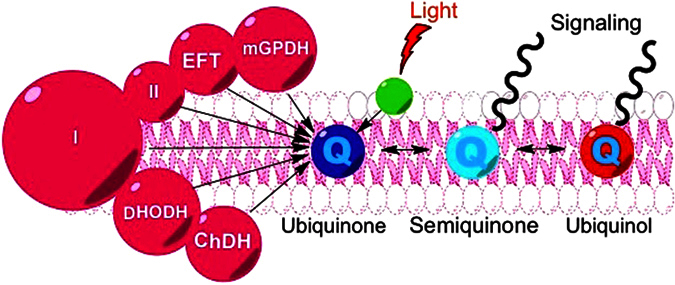
Mitochondrial CoQ_10_ photoreduction as potential mechanism to sense light. Almost all of the cell’s redox reactions culminate to reduce coenzyme Q (CoQ_10_), shown in the schematic in its three oxidations states. **I and II** (Complexes I and II): transfer electrons from NADH and FADH2, respectively, to CoQ_10_ **mGPDH** (Mitochondrial glycerol-3-phosphate dehydrogenase): oxidizes glycerol-3-phosphate to dihydroxyacetone phosphate with concurrent reduction of flavin adenine dinucleotide (FAD) to FADH_2_ and transfers electrons to CoQ_10_ **DHODH** (Dihydroorotate Dehydrogenase): catalyzes the oxidation of dihydroorotate to orotate for *de novo* pyrimidine biosynthesis, using CoQ_10_ as an electron acceptor. **ChDH** (Choline dehydrogenase): oxidizes choline to glycine-betaine with CoQ_10_ serving as the primary electron acceptor. **EFT** (Electron-transferring-flavoprotein dehydrogenase): links the oxidation of fatty acids and some amino acids to oxidative phosphorylation by the oxidation of electron-transferring-flavoprotein using CoQ_10_ as an electron acceptor. The oxidation state of the mitochondrial CoQ_10_ pool can be used as a means of signal origin. We propose that dietary chlorophyll metabolites (green circle), such as PA, can enter the mitochondria, where they can absorb long wavelength light, resulting in the photoreduction of CoQ_10_, which could then propagate the input photonic energy via several potential pathways (wavy lines).

A fundamental reaction of chlorins is the transfer of excitation energy to quinones. This energy transfer provides the initial step in the transformation of photonic energy to chemical energy in photosynthesis and thus sets the stage for life on earth. It is thus tempting to speculate on the existence of a similar process in animal cells. Data in liposomes and isolated mitochondria demonstrate that mitochondrial CoQ_10_ can be a primary recipient of PA's excitation energy leading to the reduction of CoQ_10_. CoQ_10_ photoreduction is also consistent with our observed increases in complex III activity in isolated mitochondria and subsequent increase in ATP concentrations in both isolated mitochondria and *C. elegans.* Taken together, we suggest that, in the worm, light excites PA and this excitation energy is transferred to CoQ_10_. This transfer of excitation energy from PA to CoQ_10_ could serve as the origin of a signaling cascade that results in reductions in fat mass (Fig. 6). This hypothesis was supported by experiments in which partially blocking complex III, which is upstream of CoQ_10_, with low dose antimycin A inhibited fat mass reduction upon light and PA exposure, but not upon Fluoxetine exposure. It is also in-line with the notions that the redox state of CoQ_10_ regulates mitochondrial uncoupling^26^, that there is less reduced CoQ_10_ in proportion to oxidized CoQ_10_ in adipose from obese animals^27^ and people^28^, and links between the CoQ_10_-cytochrome c reductase core protein and obesity^29^. CoQ_10_ photoreduction could provide a chemical entry point by which the long wavelength component of light and the chlorophyll component of chlorophyll-rich food may interact in the body to impact normal function.

In C. *elegans*, we used alterations in lipid as an indicator to gauge the potential significance of the absorption of additional photonic energy through the use of accessory, dietary derived pigments. The relationship between the observed phenotypes (reduced fat mass, altered lipid profiles, reduced oxidative stress, altered transcriptomes, increased ATP levels, and increased life span) may be coincidental. Further, the worm phenotypes may or may not present in higher order animals. The value of the worm is that it can be used as a simple model to suggest a likelihood that light and dietary derived pigments contribute to normal physiology. Cell types, in higher order animals, susceptible to similar perturbations would presumably be those exposed to chlorins and LWL. It has been estimated that, in humans, approximately 5% of dietary chlorophyll is absorbed^30^. Dietary derived chlorophyll pigments can be found in the liver, bile, and lining of the intestines^31^,^32^,^33^,^34^. We have shown that chlorins may also accumulate in adipose^1^. There are, however, additional routes of chlorin exposure including oral supplements and/or injections. Adipose tissue is bathed in LWL^35^,^36^,^37^. Light between 600 to 700 nm can penetrate a 1.5-inch-thick abdominal wall with three-to-five orders of magnitude of attenuation^38^,^39^. The amount of LWL in the body can also be tuned by various means ranging from bathing under sun or red light, both activities currently practiced, to wearing light-emitting diodes. There are several potential routes by which the body may be exposed to both chlorins and light to potentially photoreduce CoQ_10._

In mice, plasma α-macroglobulin is a major acute phase protein and is increased during inflammation^15^. The magnitude of reduction of α-macroglobulin was significant given that known activators of the innate immune system trigger a similar increase in α-macroglobulin^15^. The reduction in α-macroglobulin upon exposure to light and pheophytin was further significant considering we used outbread mice, in which each animal had a degree of genetic variability. Our data suggest that acute phase proteins may serve as indicators of exposure to LWL and dietary chlorophyll, and that α-macroglobulin, in particular, may be useful for quantifying this exposure in mice. As the major acute phase proteins in humans are different than those in mice, other acute phase proteins may correlate with exposure to LWL and dietary chlorophyll in humans. The phenotypic significance of a reduction in α-macroglobulin in the LWL and pheophytin treated animals, and the extent to which the relationship between the treatment and α-macroglobulin is causal or casual, remains to be elucidated.

The state of an organism is largely dictated by its environment. Elucidating these environmental determinants and how they affect function are important for establishing lifestyle recommendations to prolong human health and wellbeing. Evolutionary conserved environmental pressures – those that the body has become dependent on - provide rational starting points of inquiry. The above data suggest that, like in plants, the longer wavelength component of visible light and the chlorophyll component of chlorophyll-rich foods may act together to affect animal function, as well.

## Methods

### Photoreduction of Coenzyme Q10 in Liposome Mimics

Pheophorbide-a (PA) [Frontier Scientific, Catalog number: Pha-592, Logan, UT] (≈ 0.20 mg) and coenzyme Q [Sigma-Aldrich, Catalog number: C9538, St. Louis, MO] (50 mg) were dissolved in 1 mL of ethanol in a round bottom flask. L-α-Phosphatidylcholine [Sigma-Aldrich, Catalog number: P5638, St. Louis, MO] (112 mg), cholesterol (18 mg), Tween-80 (8 mg) and 5 mL of chloroform were then added to the flask. The solvent was removed under vacuum with a rotary evaporator at 25 °C, and trace solvent was removed by placing the flask under high vacuum for 1 hour. The flask was then charged with argon and 25 mL of saline was added. The solution was stirred at 60 °C for 1 hour to produce a suspension, which was transferred to a 50 mL conical tube, placed on ice, and then sonicated with a probe sonicator (15 seconds on/15 seconds off) for 5 minutes to yield a transparent solution. The tube was filled to 50 mL with saline, centrifuged at 11,000x g for 30 minutes, and the resulting supernatant filtered through a 0.2 μm filter. Final concentrations were: 325 μg of ubiquinone and 1.0 μg of pheophorbide-a per 500 μL of solution. The concentration of PA was measured by mixing 50 μL of the liposome in 950 μL of acetone, vortexing the solution for 1 minute, centrifugation for 5 minutes at 16,000 rpm and reading the absorbance of the supernatant at 665 nm using an extinction coefficient of 47,000 cm^−1^/M^40^. The concentration of ubiquinone was determined by UPLC as described below.

For the photoreduction, we added ascorbic acid (10 molar equivalents in comparison to CoQ_10_) to 5 mL of the liposome solution in a round bottom flask. The solution was purged with argon to prevent re-oxidation of ubiquinol, and exposed to red light from a 1.70 W, 660 nm LED light bulb. Light intensity was measured using a LI-250A light meter (LI-COR Biosciences, Lincoln, NE). At the times shown in Fig 1B, aliquots were withdrawn, mixed with 1 mL of methanol containing 1% of 80% phosphoric acid, and ubiquinol and ubiquinone were quantified by UPLC as described below.

### Quantification of ubiquinol and ubiquinone by UPLC

Stock solution of 5 mg of ubiquinone in 5 mL of butanol was prepared. To prepare ubiquinol, 1 mL of the ubiquinone stock solution was added to 100 mg of sodium borohydride. The solution was shaken for 3 minutes, 1 mL of 1 M HCL was added to decompose the unreacted sodium borohydride and the solution was centrifuged on high for one minute. 20 μL of the upper butanol layer containing ubiqunole was added to 1 mL of methanol containing 1% of 80% phosphoric acid to give a 23 pmol/mL stock solution of ubiquinol. To make a corresponding 23 pmol/mL stock solution of ubiquinone, 20 μL of the ubiquinone from 5 mg/5 mL of stock solution was added to 1 mL of the methanolic phosphoric acid. Both 23 pmol/mL stock solutions were combined and made serial dilutions down to 0.2 pmol/mL. For UPLC analysis, we injected 20 L of the above stock solutions (8 in total) into a Flexar FX-15 system with a PDA Detector. The system contained a 50 × 2.18 mm, 2.6 μm particle size, C18 column, the oven was set to 45 °C, samples were eluted isocratically with methanol:isopropanol: trifluoroacetic acid (800:200:0.1) and detected at 205 nm. The limit of detection for both ubiquinone and ubiquinol were greater than 0.004 pmol on column. The generated standard curve was used to determine ubiquinone:ubiquinol ratios in unknown sample.

### Complex III Activity

Complex III activity was measured as described by Birch-Machin and Turnbull^41^. However, using a 96-well plate format, 1,600 pmols of cytochrome c (from bovine heart; Sigma-Aldrich) per well, 20 μM pheophorbide-a, and using isolated mouse liver mitochondria^42^. For each of the shown treatment group in Fig. 1D, reactions were performed in 8 replicates. For each group, 4 of the replicates were kept in the dark and 4 were exposed to light. We used a 2-tailed, unpaired t-test to determine significance (P-values < 0.05).

### Photoreduction of Coenzyme Q10 and ATP Generation in Isolated Mitochondria

All solutions were made using type I water. All instrumentation that contacted the mitochondria or heart tissue were rinsed with type I water before use. For mitochondrial isolation, a domestic pigeon (Columbidae) was decapitated and the heart was put in an ice-cold solution of wash buffer (EDTA (0.001 M), Sucrose (0.25 M), Tris HCL (0.010 M), pH 7.4, made with type I water) within 20 seconds of death. While in the wash buffer, the heart was minced into approximately 5 mm of pieces with scissors and freed of any connective tissues. The minced heart was washed 5 times with the ice-cold wash buffer or until the buffer wash was clean, transferred into a blender tube (C Tube Miltenyi Biotec Inc. Auburn, CA) containing 10 mL of isolation buffer [D-mannitol (0.225 M), sucrose (0.075 M), EDTA (0.001 M), ATP (0.0017 M), succinate (0.002 M), glutamic acid (0.005 M), malic acid (0.0025 M), 2-amino-2-hydroxymethyl-propane-1,3-diol (0.006 M)/3-morpholinopropane-1-sulfonic acid (0.003 M), 2-mercaptoethanol (0.005 M), fatty acid free-bovine serum albumin (1 g/L), pH 7.3] and homogenized (gentleMACS™ Dissociator, program D, Miltenyi Biotec Inc. Auburn, CA). We used approximately 10 mL of isolation buffer for each gram of minced heart. The homogenate was transferred to a 50 mL tube and larger cell fragments were removed by a centrifugal spin at 600 relative centrifugal force for 10 minutes in a refrigerated centrifuge held at 4 °C. The pellet was collected and re-homogenized, as described above, in half the amount of the original buffer, and the larger cell fragments were removed from this second homogenate by another centrifugal spin at 600 relative centrifugal force for 10 minutes at 4 °C. The two supernatants were combined and mitochondria were pelleted with a 10 minute spin at 7,000 relative centrifugal force. All floating fat and fat on the side of the tube was removed. The pelleted mitochondria were washed once by re-suspending the pellet in 10 mL of reaction buffer (0.25 M Mannitol, 0.02 M HEPES, 0.01 M KCl, 0.01 M KH_2_PO_4_, 0.005 M MgAc_2_, 0.001 M EGTA, 1 mg/mL fatty acid free-BSA, pH 7.2) and centrifugation at 7,000 relative centrifugal force for 10 minutes. Between washes, the mitochondria pellets were re-suspended with 3 strokes of a custom pestle designed for the 50 mL tube.

The mitochondria pellet was suspended in 7 mL of reaction buffer. To this, we added ascorbic acid (10 mg) and ADP (20 mg) pre-dissolved in 1 mL of the reaction buffer. The suspension was divided into two equals, 4 mL of portions and 4 μL of a stock solution of PA (2.4 mg/400 μL) and pyropheophorbide-a [Frontier Scientific, Logan UT] (2.4 mg/400 μL) in dimethyl sulfoxide (DMSO) was added to one suspension to give final metabolite concentrations of 10 μM each. We added 4 μL of DMSO to the control suspension. The solutions were incubated under an atmosphere of argon, on ice and in the dark for 1 hour. We then removed the samples from the ice, replaced the atmosphere with approximately 1.5% oxygen to better simulate oxygen tensions in the mitochondria, allowed the suspensions to warm to room temperature for 10 minutes, then exposed them to 660 nm light of 4 μmol s^−1^ m^−2^.

For ATP, ADP, and AMP quantification, we withdrew 50 μL of reaction buffer, added it to a mixture of 500 μL of chloroform and 150 μL of phosphate buffer (0.1 M disodium phosphate, pH 10) and immediately vortexed the sample for 30 seconds. After all samples were collected, we centrifuged them to separate the layers, withdrew 50 μL of the aqueous layer and added it to 200 μL of a glycine buffer (0.01 M glycine, pH 10). For chromatograpic detection, 2 mL of the sample in glycine buffer was injected into the UPLC system, which was equipped with a C18, 1.7 μm particle size, 150 × 2.1 mm column held at 45 °C. We eluted at a flow of 0.7 mL per minute with a solution of potassium dihydrogen phosphate (0.107 M), tetrabutylammonium hydrogensulphate (0.0012 M) and 3.5% acetonitrile adjusted to pH 6.25 with KOH, and detected at 260 nm. For ATP, ADP, and AMP quantification, 4 point standard curves were generated for each analyte.

For detection of ATP using the luciferase assay, 5 μL of the aqueous extract was added to 50 μL of water. To this mixture, 50 μL of assay buffer was added and ATP was determined according to the manufactures instructions (CellTiter-Glo^®^ Luminescent Cell Viability, Promega, Madison, WI). For comparisons and plotting, all data was normalized to 1.

To determine the ratio of ubiquinone and ubiquinol, we withdrew 200 μL of the suspension and added to 600 μL of methanol containing 1% perchloric acid and 200 μL of heptane containing 1 mg of BHT per 10 mL, and vortexed the tube for 1 minute, centrifuged at 1,400 rpm for 5 minutes and injected 5 μL of the organic layer into the UPLC system. The UPLC conditions have been described^1^.

For protein determination, we withdrew three 50 μL aliquots from each sample, added it to 1,000 μL of water, precipitated the protein by centrifugation at 14,000 rpm for 5 minutes, washed the pellet with 1 mL of water and spun down again at 14,000 rpm. The resulting pellet was dissolved in 50 μL of 9 M urea solution, and the absorbance at 260 and 280 nm were measured (NanoVue Plus spectrophotometer, GE Healthcare Bio-Sciences, Pittsburgh, PA), and concentrations calculated with the Christian and Warburg Equation (Protein (mg/ml) = 1.55 * Abs_280_ − 0.76 * Abs_260_)^43^.

### Routine Caenorhabditis elegans maintenance

The *C. elegans* were as previously described^1^ and grown under non-sterile conditions on a substrate of an equal weight of mushrooms (*Agaricus bisporus*), bovine liver, and Red Delicious apple all blended into a fine paste with a high sheer mixer. The substrate was spread onto petri dishes, worms were added, and dishes were maintained at 25 °C under 12 hour light-dark cycles from fluorescent lights. Animals were transferred to new plates every 4 to 5 days. Rich microbial communities grew on this substrate.

### Worm synchronization

*Sucrose flotation* - Worms were washed from the above-described plates with ice-cold phosphate-buffered saline (PBS). An equal volume of the resulting worm suspension was added to an equal volume of ice-cold solution of 60% sucrose in PBS. Worms were then floated by centrifugation in a refrigerated centrifuge at 300 rpm for 3 minutes. The upper layer containing worms was removed and added to an equal volume of ice-cold PBS, the worms were pelleted by centrifugation at 3,000 rpm for 1 minute, and the pellet washed with ice-cold PBS.

For every ≈ 500 μL of worm pellet, overlaid to approximately 1 mL with saline, was added to 6 mL of a bleaching solution (1 mL of 8% sodium hypochlorite and 5 mL of 1 M sodium hydroxide) in a 50 mL conical tube. The solution was swirled for 9 minutes at room temperature, filled to 50 mL with saline, the resulting eggs pelleted with a one-minute spin, and then washed twice with saline.

### General Treatments

Substrate (0.2 g) was added to a 20 mL suspension of synchronized, one-day old worms at a density of 5 to 10 worms per microliter in S-buffer (IPM Scientific, Inc., Eldersburg, MD, Cat No.11006-505). A 7,482 μM stock solution of the PA in DMSO was prepared. From this DMSO stock, 2x concentrations of the PA in S-media were made. For the reaction, an equal volume of the 5 to 10 worms per microliter worm suspension was added to an equal volume of the 2x PA solution in an Erlenmeyer flask. For the vehicle control, DMSO was used without added PA. The resulting worm suspensions (10 mL at 2.5 to 5 worms per µL) were aerated on an orbital shaker for 1 to 6 days. For light treatments, animals were exposed to red light of 660 nm (LEDwholesalers, CA, USA) at 0.25 to 0.65 W/m^2^ for 10 hours, followed by 14 hours of darkness. For dark rearing, flasks were covered with aluminum foil. For experiments lasting past reproductive age, 5-Fluoro-2’-deoxyuridine (Sigma, St. Louis, MO, USA) was added at a concentration of 200 μM.

### Leanness measurements

Worms were imaged via light microscopy at 1,000x magnification. Widths were measured at the terminal bulb using the ImageJ software suite.

### Oil Red O staining and quantification

Each cohort of worms was collected into a 15 mL conical tube and immediately cooled on ice. The worms were pelleted by centrifugation at 3,000 rpm for 1 minute. The supernatant was withdrawn to give a 3 mL of liquid. To this, 7 mL of ice-cold isopropanol was added, to give 70% isopropanol, and the resulting worm suspensions were kept on ice for approximately 10 minutes. During this time, a pre-prepared solution of 1% solution of Oil Red O in isopropanol was mixed with water to give 0.7% Oil Red O and 70% isopropanol (i.e. 7 mL of the 1% solution with 3 mL of water). The mixture was vortexed, slightly heated, placed on ice for 10 minutes, then filtered through a 0.20 μm filter. The worms in the 70% isopropanol were pelleted with brief spin, the solution was withdrawn to 1 mL, then 5 mL of the filtered dye solution was added. The worms were kept on ice for 3 hours, then washed 3 times with water and visualized. For quantification, we measured particle density using ImageJ, 1.7 cm behind the terminal bulb.

### Biochemical Quantification of Triglycerides and Free Fatty Acids

Worms were collected and bacteria were removed by sucrose flotation. To each worm pellet in a 1.5 mL heavy walled tube, we added 0.32 g of 0.5 mm zirconium oxide beads, and 500 μL of butanol. Worms were then homogenized with use of a Bullet Blender^®^ Tissue Homogenizer (Next Advance, Inc. Averill Park, NY), setting the speed to 7 for 25 minutes. Triglycerides and free fatty acids in the resulting homogenates were quantified using commercial kits according to the manufacture’s instructions (Biovision Inc. Milpitas, CA, Catalog numbers: K622-100 for triglycerides and K612-100 for free fatty acids).

### Lipid quantification of fats by HPLC

Lipid quantification was conducted as described^44^ with modifications. Briefly, worms were collected and bacteria were removed by sucrose flotation. For lipid extraction, to each worm pellet in a 1.5 mL centrifuge tube, we added 500 μL of a modified Dole’s mixture (Isopropanol, 7.843 mL; heptane, 1.961 mL; and 2 M phosphoric acid, 0.96 mL) and homogenized the worms in the Bullet Blender^®^ as described above. Then, 400 μL of heptane and 600 μL of water were added to each tube. The tube was vortexed for 1 minute, the heptane layer was separated by centrifugation at 14,000 rpm for 5 minutes and 150 μL of heptane transferred to a screw-top tube and the heptane was evaporated under reduced pressure at 45 °C.

For free fatty acids, to each tube of evaporated heptane extract, 150 μL of derivatization buffer (p-Bromophenacylbromide, 8 mg; 18-crown-6, 0.8 mg; and 50 mL acetonitrile), 1 mg of K_2_CO_3_ and a magnetic stir bar was added. The tube was heated for 85 °C for 30 minutes while siring at 300 rpm, cooled to room temperature, centrifuged at 14,000 g for 5 minutes, and 100 μL of clear supernatant was transferred to a UPLC-injecting tube for analysis. For UPLC, we used an isocratic mobile phase of acetonitrile and water (75:25 both with 0.1% trifluoroacetic acid with a 50 × 2.18 mm, 2.6 μm particle size, C18 column.

For total extracted lipids, the lipid extract was hydrolyzed by adding 500 μL of KOH solution (KOH, 0.5 g; water, 6 mL; isopropanol, 4 mL) to each tube of the evaporated heptane extract. The tube was heated for 85 °C for 30 minutes while stirring at 300 rpm, cooled to room temperature, then 100 μL of 6 M HCl, 400 μL of water, and 300 μL of heptane were added. The sample was vortexed for 1 minute, centrifuged at 14,000 g for 5 minutes, and 200 μL of the top heptane layer was transferred to a to screw-top tube and the heptane was evaporated under reduced pressure at 45 °C. The resulting released free fatty acids were then derivatizatized as above.

### RNA Sequencing

Worms were collected and pelleted by centrifugation in a 1.5 mL tube. To the pellet, we added 500 μL of RNAlater§ vortexed to disrupt the pellet, and kept the samples at 4 °C for overnight. Worms were pelleted by centrifugation, the RNA latter was removed, the worms were homogenized in a Bullet Blender^®^ Tissue Homogenizer as previously described in a commercial homogenization buffer (RNeasy Mini Kit, Qiagen, Valencia, CA) and total RNA was isolated according to the manufacture’s instructions. RNA sequencing and bioinformatics were performed by the Otogenetics Corporation (Norcross, GA, Catalog numbers: Oto-Rseq2-20 and Oto-Rseq-Bio1).

### Lipid Carbonyls

Was performed according to literature procedures^45^,^46^ with the below modifications. To a 1.5 mL screw-top tube, we added 200 μL of the worm homogenate, 25 μL of thiobarbituric acid solution (thiobarbituric acid, 0.8 g; 0.1 M sodium hydroxide, 50 mL; 25 μL of butylated hydroxytoluene solution (71.4 mg of butylated hydroxytoluene in 1.5 mL of ethanol) and 200 μL of 0.2 M phosphoric acid). The reaction was heated for 45 minutes at 90 °C then cooled to room temperature with an ice bath. To each tube, we then added 500 μL of butanol containing 5% pyridine and 50 μL of a saturated sodium chloride solution. We vortexed each tube for 1 minute, centrifuged at 12,000 rpm for 5 minutes and transferred 250 μL of upper butanol phase into flat-bottom 96-well plate and measured fluorescence using 540 nm excitation and 590 nm emission. Total protein was assayed in the original worm homogenate sample by UV-vis spectroscopy as described above.

### Protein Carbonyls

Was performed according to literature procedures^47^ with the below modifications. Worms were collected, bacteria were removed by sucrose flotation, and worms homogenized in PBS using a Bullet Blender^®^, as described above. To a 2 mL centrifuge tube, we added 200 μL of worm homogenate (at protein concentration was about 10 μg per μL of homogenate) and 200 μL of 0.2 mM of fluorescein thiosemicarbazide (FTC). The tubes were incubated at room temperature on a shaker for 24 hours in the dark. To each tube, we then added 1.6 mL of ice-cold 20% trichloroacetic acid, incubated the solution for 10 minutes on ice to precipitate protein, and pelleted the protein by centrifugation at 12,000 rpm at 4 °C for 10 minutes. The supernatant was aspirated and discarded, and the pellet was washed 3 times with 1 mL of acetone at 4 °C until no fluorescence signal, at 495 nm excitation and 535 nm emission, was detected in the acetone wash. For washing, the pellet was dislodged during each wash and re-pelleted by centrifugation at 12,000 rpm at 4 °C for 10 minutes. The resulting pellet was air dried and dissolved in 300 μL of 6 M guanidine hydrochloride. The resulting labeled protein carbonyls were measured by placing 250 μL into a flat-bottom 96-well plate and measuring fluorescence using 495 nm excitation and 535 nm emission. Total protein was assayed in the labeled sample by UV-vis spectroscopy as described above.

### Life Spans

To a 25 mL Erlenmeyer flask, was added 10 mL of synchronized L1 worms at concentration of 300 worms/mL, 25 μL of food substrate (to give an OD_600_ = 2.5), 200 μL of a 100 × penicillin/streptomycin (Amresco, Solon, OH, USA) solution in S-media, and 10 μL of a 5 mg/mL amphotericin B (Amresco, Solon, OH, USA) solution in S-media. The resulting worm suspension (100 μL) was added to each well of a 96-well plate, the plates were wrapped with paraffin to prevent evaporation, and then incubated at 20 °C on an orbital shaker at a speed of 110 rpm. At 1 day of age, we added 100 μL of a PA stock solution (24 μM of PA with 0.16% DMSO in S-media) or DMSO vehicle to each well. We used 8 replicates or 8 wells for each cohort. At 2.5 days of age, to each well, we added 10 μL of a 4.2 mM solution of FUdR in S-media. Beginning at 5 days of age, the worms were exposed to LWL (660 nm at 0.25 W/m^2^, 5 h per day light and 19 hours darkness) or kept in the dark by covering the plate with aluminum foil. Worms were counted and scored dead or alive every 2 or 3 days until all worms were dead.

### Food Consumption

We followed literature methods^48^ with the following modifications. The food substrate was sonicated with a probe sonicator and filtered through a 8.0 μm millicell filter (Millipore, Billerica, MA, USA) to give a homogenous solution. To 2 mL of the homogenous food substrate, we then added 14 μL of 7,482 μM PA stock solution in DMSO or DMSO vehicle. Worms were harvested and bacteria were removed through gradient sucrose (30% and 15%). To each well of a 24 well plate, we added 20 μL of food substrate (containing either PA or DMSO vehicle), 3 μL of a 10 × penicillin/streptomycin stock solution in S-media, and 23 μg/mL of an amphotericin B stock solution in S-media, and 5 μL of a 60 worms/μL of worm suspension. The plates were wrapped with paraffin and placed on shaker at a speed of 110 rpm, and exposed to LWL (0.5 W/m^2^, 10 hours per day light and 14 hours darkness). For food quantification, 300 μL of a 9 M urea buffer was added to each well to lyse the food substrate (the urea did not lyse the worms). Then, 250 μL of the resulting solution was added to a 96-well plate and the absorbance at 500 nm was measured. The absorbance at 500 nm was dependent on the concentration of the food substrate and neither the worms, nor the PA, significantly contributed to the absorbance at 500 nm (Fig. S7).

### Reproduction

PA, in a stock solution in DMSO, was mixed with food substrate to give a concentration of 25 μM PA. 50 μL of this substrate or 50 μL or substrate without PA, but balanced in DMSO, were added to each well of a 96-well plate, followed by a 2 μL suspension of L1 worms in S-media, to give approximately 5 worms per well. The plate was covered with a clear plate cover and placed under LWL of 0.35 W/m^2^ for 6 h per day. For the dark groups, aluminum foil was used to block the light. At the times shown, 100 μL of PBS where added to each well, the number of adult worms per well along with all offspring and eggs were counted in 10 μL aliquots from each well.

### Nile Red Staining

A 150 μL suspension of L1 worms in S-media containing heat inactivated *E. coli K-12* was placed into each well of a 96-well plate followed by a solution of Nile red, to give a 150 nM final concentration of Nile red, and PA or DMSO vehicle. 32 replicates were used for each group. The plate was put onto a shaker and exposed to 0.25 W/m^2^ LWL for 6 h per day for 3 days. Nile red fluorescence (Ex/Em = 485/535 nm) from each well was then measured with use of a plate reader.

### *In vivo* ATP

To a 50 mL Erlenmeyer flask was added, 30 mL of synchronized L1 worms at concentration of 15,000 worms/mL, and 75 μL of food substrate (to give an OD_600_ of 2.5). Then to each well of a 12-well plate was added, 2 mL of the above worm suspension, and either 500 μL of a 1 mM fluoxetine solution in S-media, 8.5 μL of 7,482 μM PA stock solution in DMSO, or DMSO vehicle (all reaction contained the same volume of media and DMSO). We used 6 replicates for each cohort. The worms solutions were then incubated in dark for 2 days on orbital shaker at a speed of 110 rpm. For ATP measurement, 80 μL of worm suspension was pipetted into each well of a 96-well plate and the worms were exposed to LWL (0.25 W/m^2^) for 30 minutes. Then, 80 μL of a 2 × luciferin buffer (citrate phosphate buffer, pH 6.5, plus 1% DMSO, 0.05% Triton X-100, and 100 μM D-luciferin) was quickly added to each well, the plate agitated to mix the solution, and incubated under the LWL for an additional 25 minutes before recording the luminesces signal from each well in a plate reader.

### Mice

All experiments were conducted in accordance with the current *Guide for the Care and Use of Laboratory Animals*, published by the US National Academies Press. Columbia University’s Institutional Animal Care and Use Committee (IACUC) approved animal protocols. Four week old, male mice (SKH1-Elite Mouse, Crl:SKH1-Hrhr, Charles River) were fed a purified diet (Product# F3282, Bioserve, Flemington, NJ). Pheophytin was isolated from chlorella according to standard methods^49^ and was administered in the drinking water at a concentration of 1 mg of pheophytin per mL water. The water or liquid diet was prepared by mixing 900 mL water, 300 g sucrose, 50 mL vegetable oil, 5 g sucrose fatty acid esters, and 1 g pheophytin in a high sheer mixture. Pheophytin naïve animals received the same diet without added pheophytin. Animals were housed under either yellow (580 nm) LED light or an incandescent light. The light intensities at the bottom of the cages were between 2 and 5 μmol s^−1^ m^−2^. Otherwise, all animals were kept as described in the “Guide for the Care and Use of Laboratory Animals”. Blood was collected via submandibular bleeding. A MILLIPLEX^®^ MAP Magnetic Bead Panel (EMD Millipore Corporation., Darmstadt, Germany) was used to determine all acute phase proteins.

## Additional Information

**How to cite this article**: Zhang, D. *et al*. Sequestration of ubiquitous dietary derived pigments enables mitochondrial light sensing. *Sci. Rep.* **6**, 34320; doi: 10.1038/srep34320 (2016).

## Supplementary Material

Supplementary Information
