# Supplementary material for: Sequestration of ubiquitous dietary derived pigments enables mitochondrial light sensing
Source: Sci Rep. 2016 Oct 12;6:34320. doi: 10.1038/srep34320 (PMC5059631; doi:10.1038/srep34320)
Supplement: Supplementary Information [file srep34320-s1.pdf]

Sequestration of ubiquitous dietary derived pigments enables mitochondrial light sensing

Dan Zhang, Doina M. Mihai, Kiera Robinson, and Ilyas Washington*

Columbia University Medical Center, Ophthalmology, New York, NY 10032, USA.
*Correspondence: iw2101@columbia.edu

SUPPLEMENTAL INFORMATION

SUPPLEMENTAL FIGURES

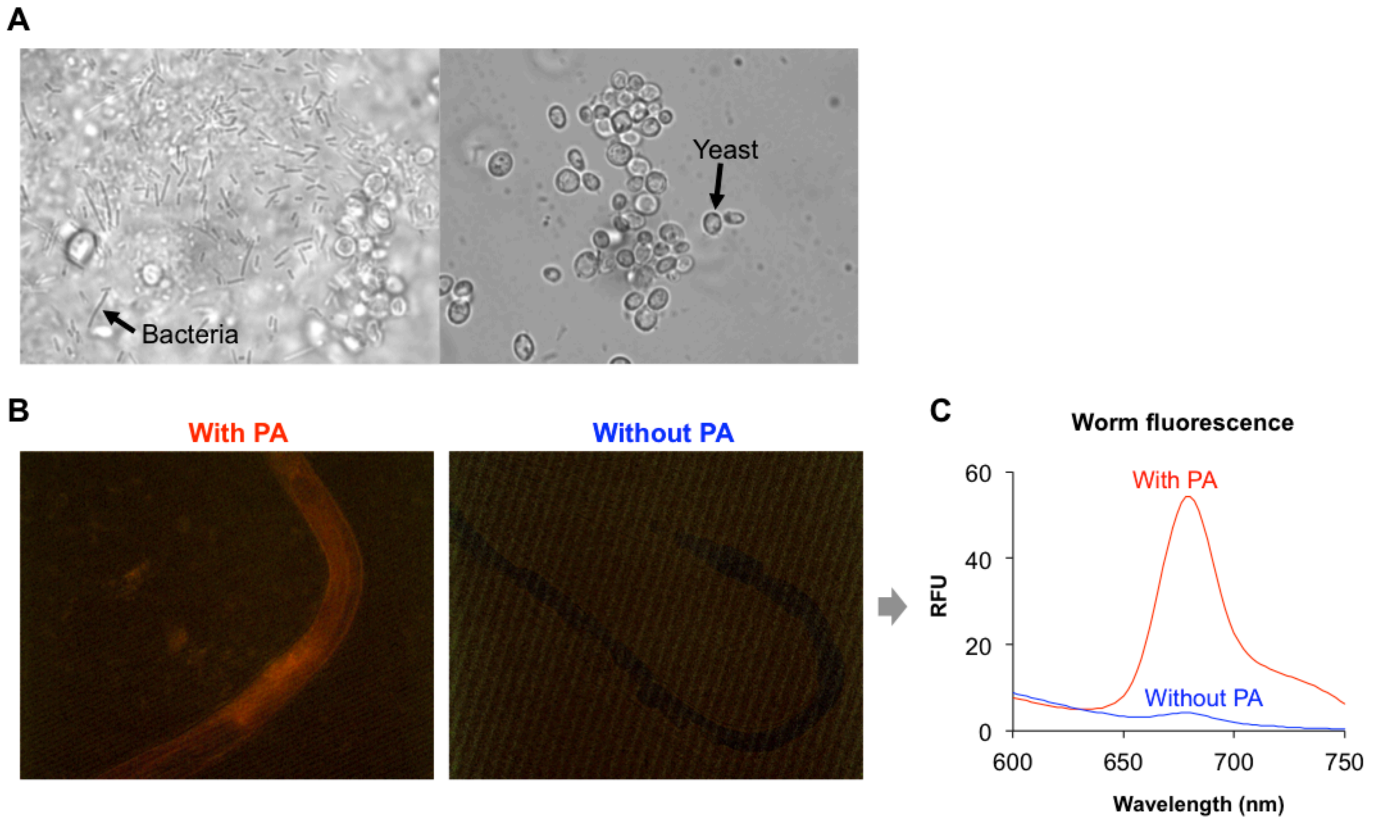

Supplementary Figure 1.
(A) Wild worm cultures. Representative images of microbial communities taken at 1000 x magnification. To more closely mimic the worm's natural habitats, worms were raised under non-sterile conditions on a substrate of mushrooms, apples and bovine liver (see experimental section), producing cultures rich in bacteria/fungi.
(B) Worms readily took up PA. Representative fluorescence image, taken with 400 nm excitation and collecting light above 600 nm, of an animal co-incubated with PA for 12 hours compared with an image of a PA naïve animal taken under identical acquisition conditions.
(C) Fluorescence spectra of the same PA treated and PA naïve worms, with 410 nm excitation. Red 665 nm fluorescence is indicative of PA.

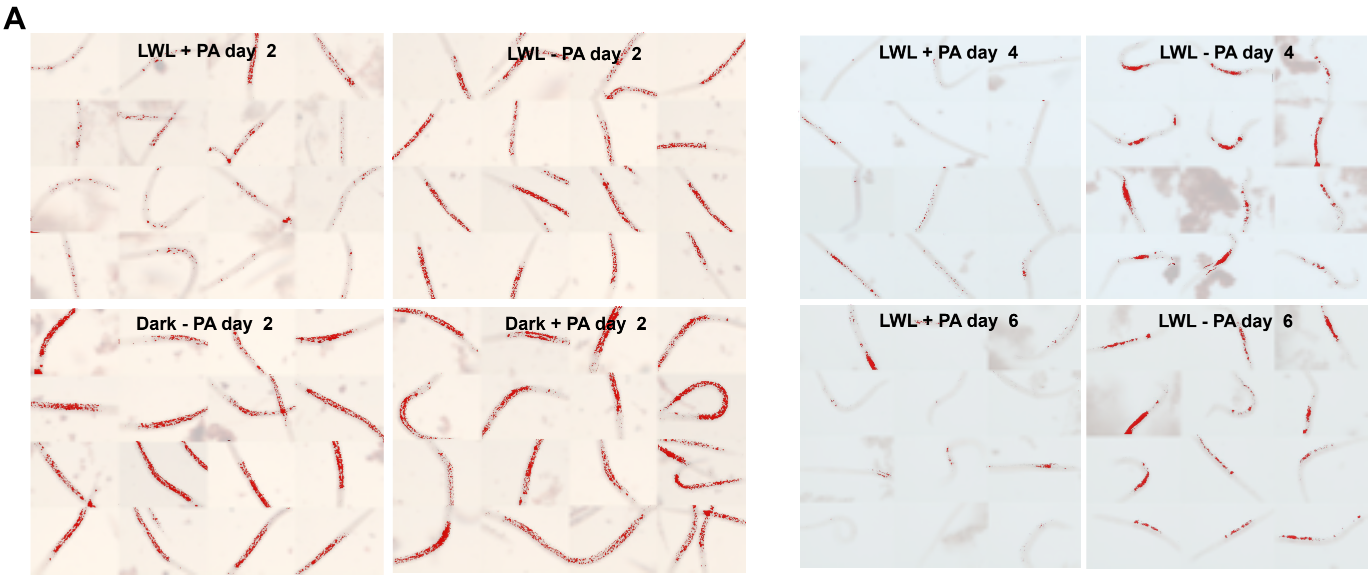

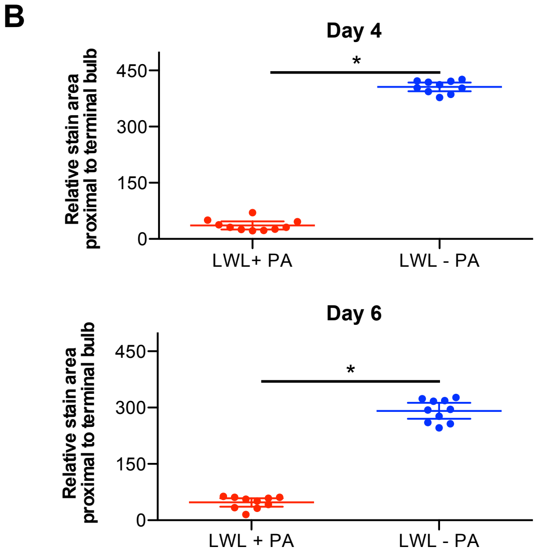

Table S1. Oil Red O quantification of body fat			
Age (day)	LWL + PA vs. LWL - PA	LWL + PA vs. Dark - PA	LWL + PA vs. Dark + PA
2	89 ± 24 vs. 410 ± 18<br>Decrease: -78%<br>P<0.0001	89± 24 vs. 409 ± 17<br>Decrease : -78%<br>P<0.0001	89± 24 vs. 404 ± 19<br>Decrease : -78%<br>P<0.0001
4	36 ± 15 vs. 407 ± 16<br>Decrease : -91%<br>P<0.0001		
6	48 ± 16 vs. 292 ± 30<br>Decrease : -84%<br>P<0.0001		

Supplementary Figure 2. Environmental lighting regulates body fat in *C. elegans, expanded.*
(**A**) Oil Red O staining highlighting fat stores. Representative images of worms raised on wild bacteria/fungi under cyclic light in the presence of PA (skinny worms) compared to control worms. Images have been processed for Oil Red O quantification.
(**B**) Oil Red O quantification for animals raised under cyclic light in the presence or absence of PA at 4 and 6-day of age. Note, because Oil Red O penetrates older worms to a lesser extent, comparisons cannot be made between worms of different ages. *: P-value < 0.05 determined by unpaired Student's t-test.

Supplementary Table 1. Numerical summary of Oil Red O quantification as shown in Figure 2C of the Results section and panels *A* and *B* in Supplement Figure 2.

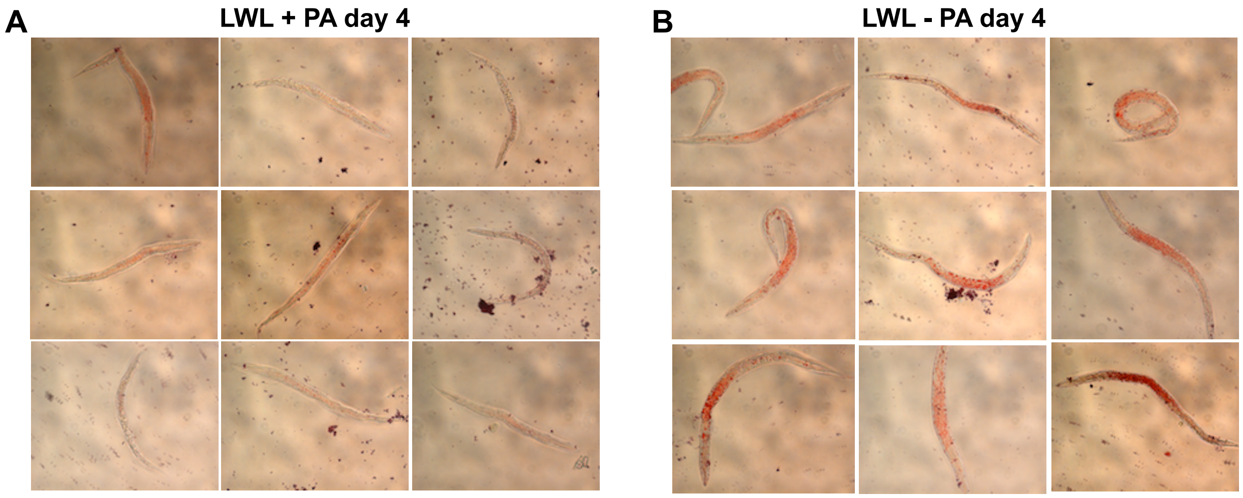

Supplementary Figure 3. Oil Red O staining highlighting fat stores. Representative images of worms raised on wild bacteria/fungi under cyclic light in the presence of PA (skinny worms) compared to control, PA naïve worms raised identically. Worms raised on mushroom substrate responded similarly to worms raised on the mixed substrate of mushrooms, apples and bovine liver.

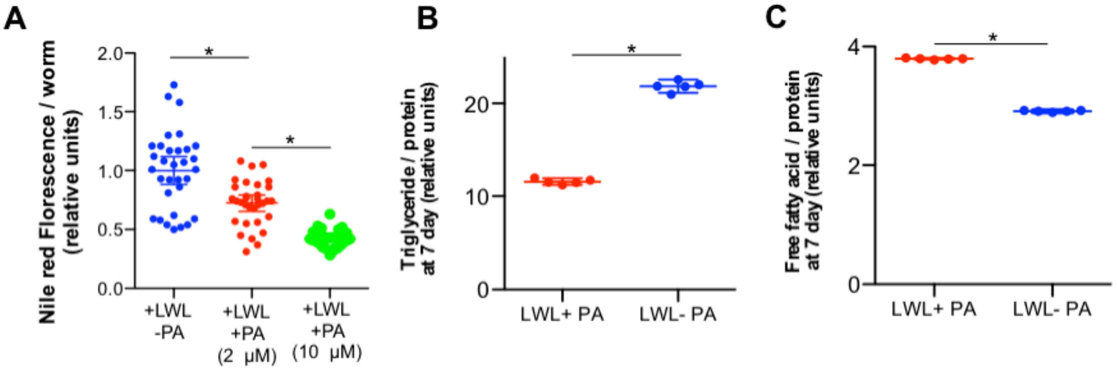

Supplementary Figure 4. Worms raised on *E. coli.* K-12 responded similarly to worms raised on wild bacteria and fungus.

(A) Quantification of Nile red fluorescence in worms exposed to LWL in the presence and absence of PA using *E. coli* K-12 as a substrate. Averages and standard deviations are shown. Each data point represents one well of a 96-well plate. Worms were treated from the L1 stage to 3 days of age.

(B) Triglycerides quantification in light exposed animals in the presence or absence of PA, using *E. coli* K-12 as a substrate, at 7 days of treatment and age. Replicate measures are shown with averages and 95% confidence intervals. *: P-value < 0.05 determined by unpaired, two sided, Student's t-test.

(C) Total free fatty acids in worms described in panel B.

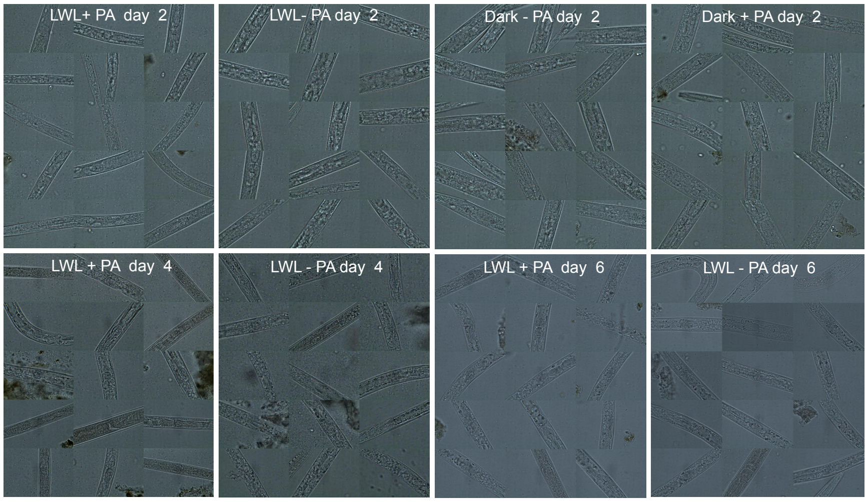

Table S2. Worm thickness at terminal bulb

Age (day)	LWL + PA vs. LWL - PA	LWL + PA vs. Dark - PA	LWL + PA vs. Dark + PA
2	1.3 ± 0.066 vs.<br>1.6 ± 0.073<br>Decrease: -19%<br>P < 0.0001	1.3 ± 0.066 vs.<br>1.7 ± 0.057<br>Decrease : -24%<br>P < 0.0001	1.3 ± 0.066 vs.<br>1.7 ± 0.049<br>Decrease : -24%<br>P < 0.0001
4	2.6 ± 0.23 vs.<br>2.9 ± 0.19<br>Decrease: -10%<br>P= 0.0016	2.6 ± 0.23 vs.<br>2.8 ± 0.19<br>Decrease : - 7%<br>P= 0.03	2.6 ± 0.23 vs.<br>2.9 ± 0.21<br>Decrease : -10%<br>P= 0.035
6	2.8 ± 0.072 vs.<br>3.0 ± 0.12<br>Decrease: -7%<br>P= 0.0008	2.8 ± 0.072 vs.<br>3.1 ± 0.17<br>Decrease: -10%<br>P= 0.025	2.8 ± 0.072 vs.<br>3.0 ± 0.099<br>Decrease: -7%<br>P= 0.0007

Supplementary Figure 5. Environmental lighting regulates leanness in *C. elegans, expanded.* Representative images of worms raised on wild bacteria/fungi under cyclic light in the presence of PA (lean worms) compared to control worms raised on the same diet but under the conditions shown in the figure caption 2F.

Supplementary Table 2. Numerical summary of worm thickness measured at the terminal bulb for animals raised under the described conditions. P value determined by unpaired Student’s t-test.

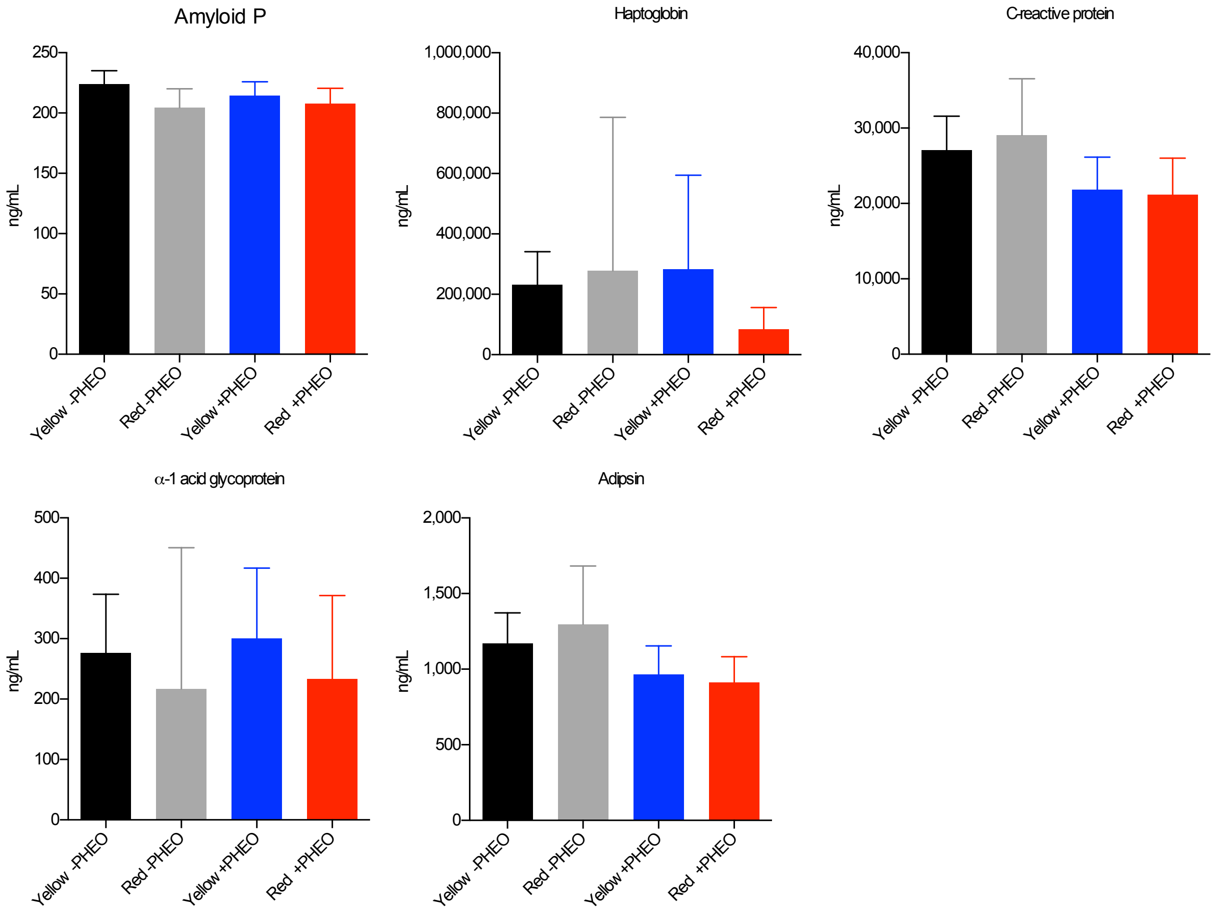

Supplementary Figure 6. Acute phase proteins in mice after 4 months of treatment. Averages and 95% confidence intervals are shown.

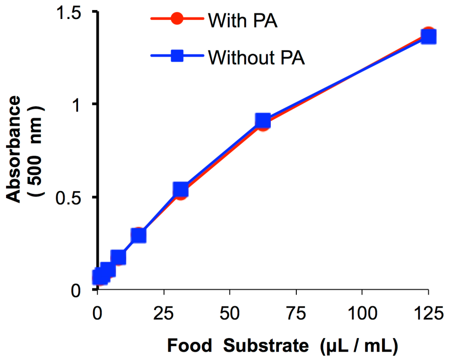

	Equation	R^2
+PA	$y = 0.0473\ x^{0.6875}$	0.99
-PA	$y = 0.0512\ x^{0.6707}$	0.99

Supplementary Figure 6. Representative absorbances of solutions of varying concentrations of food substrate with or without the same concentration of PA.

STATISTICAL ANALYSIS

Figure 2A

Groups	P value (t-test)
+LWL-PA & +Dark-PA	0.75
+LWL-PA & +LWL+ Fluoxetine	0.18
+LWL-PA & +Dark+ Fluoxetine	0.45
+LWL-PA & +LWL+PA	0.00033
+LWL-PA & +Dark+PA	0.67
+Dark-PA & +LWL+ Fluoxetine	0.30
+Dark-PA & +Dark+ Fluoxetine	0.61
+Dark-PA & +LWL+PA	0.0012
+Dark-PA & +Dark+PA	0.87
+LWL+Prozac & +Dark+ Fluoxetine	0.74
+LWL+ Fluoxetine & +LWL+PA	0.016
+LWL+ Fluoxetine & +Dark+PA	0.46
+Dark+ Fluoxetine & +LWL+PA	0.026
+Dark+ Fluoxetine & +Dark+PA	0.74
+LWL+PA & +Dark+PA	0.0061

Figure 2C

Table Analyzed	2 days				
Repeated Measures ANOVA					
P value	< 0.0001				
P value summary	****				
Are means signif. different? (P < 0.05)	Yes				
Number of groups	4				
F	753.4				
R square	0.9882				
Was the pairing significantly effective?					
R square	0.005872				
F	1.501				
P value	0.1978				
P value summary	ns				
Is there significant matching? (P < 0.05)	No				
ANOVA Table	SS	df	MS		
Treatment (between columns)	4.541	3	1.514		
Individual (between rows)	0.02714	9	0.003016		
Residual (random)	0.05424	27	0.002009		
Total	4.622	39			
Bonferroni's Multiple Comparison Test	Mean Diff.	t	Significant? P < 0.05?	Summary	95% CI of diff
+LWL+PA vs +LWL-PA	-0.7810	38.96	Yes	****	-0.8381 to -0.7239
+LWL+PA vs +Dark-PA	-0.7830	39.06	Yes	****	-0.8401 to -0.7259
+LWL+PA vs +Dark+PA	-0.7700	38.41	Yes	****	-0.8271 to -0.7129
+LWL-PA vs +Dark-PA	-0.002000	0.09978	No	ns	-0.05906 to 0.05506
+LWL-PA vs +Dark+PA	0.01100	0.5488	No	ns	-0.04606 to 0.06806
+Dark-PA vs +Dark+PA	0.01300	0.6486	No	ns	-0.04406 to 0.07006

Figure 2D

Table Analyzed	0.5			
Two-way RM ANOVA	Matching by cols			
Source of Variation	% of total variation	P value		
Interaction	21.33	< 0.0001		
Time	22.90	< 0.0001		
Column factor	32.93	< 0.0001		
Subjects (matching)	7.2986	0.5463		
Source of Variation	P value summary	Significant?		
Interaction	****	Yes		
Time	****	Yes		
Column factor	****	Yes		
Subjects (matching)	ns	No		
Source of Variation	Df	Sum-of-squares	Mean square	F
Interaction	6	0.06785	0.01131	9.150
Time	2	0.07285	0.03643	29.48
Column factor	3	0.1047	0.03492	30.08
Subjects (matching)	20	0.02322	0.001161	0.9393
Residual	40	0.04943	0.001236	
Number of missing values	0			
Bonferroni multiple comparisons	Number of comparisons: 9			
LWL-PA no worm vs LWL-PA				
Column factor	LWL-PA no worm	LWL-PA	Difference	95% CI of diff.
0.0	1.003	1.003	0.0	-0.05780 to 0.05780
2.000	1.003	0.9200	-0.08333	-0.1411 to -0.02554
5.000	0.9967	0.8583	-0.1383	-0.1961 to -0.08054
Column factor	Difference	t	P value	Summary
0.0	0.0	0.0	P > 0.05	ns
2.000	-0.08333	4.148	P < 0.001	***
5.000	-0.1383	6.886	P < 0.0001	****
LWL-PA no worm vs LWL+PA				
Column factor	LWL-PA no worm	LWL+PA	Difference	95% CI of diff.
0.0	1.003	0.9950	-0.008333	-0.06613 to 0.04946
2.000	1.003	0.9217	-0.08167	-0.1395 to -0.02387
5.000	0.9967	0.8350	-0.1617	-0.2195 to -0.1039
Column factor	Difference	t	P value	Summary
0.0	-0.008333	0.4148	P > 0.05	ns
2.000	-0.08167	4.065	P < 0.01	**
5.000	-0.1617	8.047	P < 0.0001	****
LWL-PA no worm vs LWL+PA no worm				
Column factor	LWL-PA no worm	LWL+PA no worm	Difference	95% CI of diff.
0.0	1.003	0.9950	-0.008333	-0.06613 to 0.04946
2.000	1.003	0.9950	-0.008333	-0.06613 to 0.04946
5.000	0.9967	0.9950	-0.001667	-0.05946 to 0.05613
Column factor	Difference	t	P value	Summary
0.0	-0.008333	0.4148	P > 0.05	ns
2.000	-0.008333	0.4148	P > 0.05	ns
5.000	-0.001667	0.08296	P > 0.05	ns

Figure 2G

Table Analyzed	2 days				
Repeated Measures ANOVA					
P value	< 0.0001				
P value summary	****				
Are means signif. different? (P < 0.05)	Yes				
Number of groups	3				
F	25.75				
R square	0.5754				
Was the pairing significantly effective?					
R square	0.06252				
F	0.3142				
P value	0.9956				
P value summary	ns				
Is there significant matching? (P < 0.05)	No				
ANOVA Table	SS	df	MS		
Treatment (between columns)	0.7962	2	0.3981		
Individual (between rows)	0.09227	19	0.004856		
Residual (random)	0.5874	38	0.01546		
Total	1.476	59			
Bonferroni's Multiple Comparison Test	Mean Diff.	t	Significant? P < 0.05?	Summary	95% CI of diff
+LWL-PA vs +LWL+PA	0.1907	4.851	Yes	****	0.09224 to 0.2892
+LWL-PA vs +LWL+ Fluoxetine	0.2754	7.006	Yes	****	0.1770 to 0.3739
+LWL+PA vs +LWL+ Fluoxetine	0.08474	2.155	No	ns	-0.01374 to 0.1832

Figure 2H

Table Analyzed	2 days				
Repeated Measures ANOVA					
P value	< 0.0001				
P value summary	****				
Are means signif. different? (P < 0.05)	Yes				
Number of groups	3				
F	145.7				
R square	0.9123				
Was the pairing significantly effective?					
R square	0.02693				
F	0.6313				
P value	0.8164				
P value summary	ns				
Is there significant matching? (P < 0.05)	No				
ANOVA Table	SS	df	MS		
Treatment (between columns)	5.791	2	2.896		
Individual (between rows)	0.1757	14	0.01255		
Residual (random)	0.5565	28	0.01988		
Total	6.523	44			
Bonferroni's Multiple Comparison Test	Mean Diff.	t	Significant? P < 0.05?	Summary	95% CI of diff
+LWL- PA vs +LWL+ PA	0.7000	13.60	Yes	****	0.5689 to 0.8311
+LWL- PA vs +LWL- Fluoxetine	0.8100	15.73	Yes	****	0.6789 to 0.9411
+LWL+ PA vs +LWL- Fluoxetine	0.1100	2.137	No	ns	-0.02109 to 0.2411

Figure 2I

Table Analyzed	2 days				
Repeated Measures ANOVA					
P value	< 0.0001				
P value summary	****				
Are means signif. different? (P < 0.05)	Yes				
Number of groups	3				
F	47.33				
R square	0.7593				
Was the pairing significantly effective?					
R square	0.1242				
F	1.179				
P value	0.3383				
P value summary	ns				
Is there significant matching? (P < 0.05)	No				
ANOVA Table	SS	df	MS		
Treatment (between columns)	0.3684	2	0.1842		
Individual (between rows)	0.06884	15	0.004589		
Residual (random)	0.1168	30	0.003893		
Total	0.5541	47			
Bonferroni's Multiple Comparison Test	Mean Diff.	t	Significant? P < 0.05?	Summary	95% CI of diff
+LWL-PA+Antimycin A vs +LWL+ PA + Antimycin A	0.02944	1.335	No	ns	-0.02649 to 0.08538
+LWL-PA+Antimycin A vs +LWL+ Fluoxetine + Antimycin A	0.1988	9.013	Yes	****	0.1429 to 0.2548
+LWL+PA+Antimycin A vs +LWL+ Fluoxetine + Antimycin A	0.1694	7.678	Yes	****	0.1134 to 0.2253

Figure 3A

Group	P value (t-test)
+LWL+PA & +LWL-PA	0.0073

Figure 3B

Group	P value (t-test)
+LWL+PA & +LWL-PA	0.0037

Figure 4A

Best-fit values	
Slope	-0.01 ± 0.004
Y-intercept when X=0.0	1 ± 0.05
X-intercept when Y=0.0	103
1/slope	-103
95% Confidence Intervals	
Slope	-0.02 to -0.0006
Y-intercept when X=0.0	0.9 to 1
X-intercept when Y=0.0	57 to 1544
Goodness of Fit	
R square	0.4
Sy.x	0.1
Is slope significantly non-zero?	
F	6
DFn, DFd	1, 10
P value	0.0390
Deviation from zero?	Significant
Data	
Number of X values	4
Maximum number of Y replicates	3
Total number of values	12
Number of missing values	4

Figure 4B

Table Analyzed	Data 1
Column A	+LWL-PA
vs	vs
Column B	+LWL+PA
Paired t test	
P value	< 0.0001
P value summary	****
Are means signif. different? (P < 0.05)	Yes
One- or two-tailed P value?	Two-tailed
t, df	t=17.15 df=5
Number of pairs	6
How big is the difference?	
Mean of differences	0.8083
95% confidence interval	0.6871 to 0.9295
R square	0.9833

Figure 4C

Table Analyzed	Worm producing			
Two-way ANOVA	Matching by cols			
Source of Variation	% of total variation	P value		
Interaction	0.24	0.8311		
Time	72.95	< 0.0001		
Column factor	3.07	0.0657		
Subjects (matching)	14.0571	0.1347		
Source of Variation	P value summary	Significant?		
Interaction	ns	No		
Time	****	Yes		
Column factor	ns	No		
Subjects (matching)	ns	No		
Source of Variation	Df	Sum-of-squares	Mean square	F
Interaction	3	53.20	17.73	0.2916
Time	1	16474	16474	270.9
Column factor	3	693.0	231.0	2.619
Subjects (matching)	36	3175	88.18	1.450
Residual	36	2189	60.81	
Number of missing values	0			
Bonferroni multiple comparisons	Number of comparisons: 6			
LWL+ PA vs LWL- PA				
Column factor	LWL+ PA	LWL- PA	Difference	95% CI of diff.
4	22.50	18.60	-3.900	-14.37 to 6.572
5	52.80	46.70	-6.100	-16.57 to 4.372
Column factor	Difference	t	P value	Summary
4	-3.900	1.010	P > 0.05	ns
5	-6.100	1.580	P > 0.05	ns
LWL+ PA vs Dark - PA				
Column factor	LWL+ PA	Dark - PA	Difference	95% CI of diff.
4	22.50	27.40	4.900	-5.572 to 15.37
5	52.80	53.70	0.9000	-9.572 to 11.37
Column factor	Difference	t	P value	Summary
4	4.900	1.269	P > 0.05	ns
5	0.9000	0.2332	P > 0.05	ns
LWL+ PA vs Dark + PA				
Column factor	LWL+ PA	Dark + PA	Difference	95% CI of diff.
4	22.50	23.80	1.300	-9.172 to 11.77
5	52.80	53.90	1.100	-9.372 to 11.57
Column factor	Difference	t	P value	Summary
4	1.300	0.3368	P > 0.05	ns
5	1.100	0.2850	P > 0.05	ns

Figure 4D

	LWL+PA	LWL-PA	Dark-PA	Dark+PA	Global (shared)
Null hypothesis					LogIC50 same for all data sets
Alternative hypothesis					LogIC50 different for each data set
P value					< 0.0001
Conclusion (alpha = 0.05)					Reject null hypothesis
Preferred model					LogIC50 different for each data set
F (DFn, DFd)					29.84 (3,440)
LogIC50 different for each data set					
Best-fit values					
Bottom	= 0.0	= 0.0	= 0.0	= 0.0	
Top	= 1.000	= 1.000	= 1.000	= 1.000	
LogIC50	17.72	16.05	14.94	15.35	
HillSlope	-0.1371	-0.1571	-0.1426	-0.1922	
IC50	5.220e+017	1.119e+016	8.741e+014	2.214e+015	
Span	= 1.000	= 1.000	= 1.000	= 1.000	
Std. Error					
LogIC50	0.2084	0.1510	0.2968	0.1867	
HillSlope	0.008058	0.007215	0.01125	0.01287	
95% Confidence Intervals					
LogIC50	17.30 to 18.13	15.75 to 16.35	14.35 to 15.53	14.97 to 15.72	
HillSlope	-0.1531 to -0.1211	-0.1714 to -0.1428	-0.1649 to -0.1203	-0.2178 to -0.1667	
IC50	2.015e+017 to 1.352e+018	5.617e+015 to 2.231e+016	2.253e+014 to 3.391e+015	9.436e+014 to 5.193e+015	
Goodness of Fit					
Degrees of Freedom	110	110	110	110	
R square	0.9553	0.9766	0.9191	0.9613	
Absolute Sum of Squares	0.8331	0.4757	1.659	0.8427	
Sy.x	0.08702	0.06576	0.1228	0.08753	
Constraints					
Bottom	Bottom = 0.0	Bottom = 0.0	Bottom = 0.0	Bottom = 0.0	
Top	Top = 1.000	Top = 1.000	Top = 1.000	Top = 1.000	
LogIC50 same for all data sets					
Best-fit values					
Bottom	= 0.0	= 0.0	= 0.0	= 0.0	
Top	= 1.000	= 1.000	= 1.000	= 1.000	
LogIC50	15.95	15.95	15.95	15.95	
HillSlope	-0.1303	-0.1570	-0.1418	-0.1953	
IC50	8.859e+015	8.859e+015	8.859e+015	8.859e+015	
Span	= 1.000	= 1.000	= 1.000	= 1.000	
Std. Error					
LogIC50	0.1175	0.1175	0.1175	0.1175	
HillSlope	0.008315	0.01109	0.009471	0.01553	
95% Confidence Intervals					
LogIC50	15.72 to 16.18	15.72 to 16.18	15.72 to 16.18	15.72 to 16.18	
HillSlope	-0.1466 to -0.1140	-0.1787 to -0.1353	-0.1604 to -0.1233	-0.2257 to -0.1648	
IC50	5.213e+015 to 1.505e+016	5.213e+015 to 1.505e+016	5.213e+015 to 1.505e+016	5.213e+015 to 1.505e+016	
Goodness of Fit					
Degrees of Freedom					
R square	0.9274	0.9765	0.9108	0.9575	
Absolute Sum of Squares	1.354	0.4776	1.830	0.9240	

Sy.x					
Constraints					
Bottom	Bottom = 0.0	Bottom = 0.0	Bottom = 0.0	Bottom = 0.0	
Top	Top = 1.000	Top = 1.000	Top = 1.000	Top = 1.000	
LogIC50	LogIC50 is shared	LogIC50 is shared	LogIC50 is shared	LogIC50 is shared	
Number of points					
Analyzed	112	112	112	112	

Figure S4A

Table Analyzed	Data 1				
Repeated Measures ANOVA					
P value	< 0.0001				
P value summary	****				
Are means signif. different? (P < 0.05)	Yes				
Number of groups	3				
F	63.76				
R square	0.6729				
Was the pairing significantly effective?					
R square	0.2027				
F	1.554				
P value	0.0703				
P value summary	ns				
Is there significant matching? (P < 0.05)	No				
ANOVA Table	SS	df	MS		
Treatment (between columns)	5.386	2	2.693		
Individual (between rows)	2.035	31	0.06563		
Residual (random)	2.619	62	0.04223		
Total	10.04	95			
Bonferroni's Multiple Comparison Test	Mean Diff.	t	Significant? P < 0.05?	Summary	95% CI of diff
+LWL-PA vs +LWL+PA 2 μM	0.2769	5.389	Yes	****	0.1505 to 0.4033
+LWL-PA vs +LWL+PA 10 μM	0.5800	11.29	Yes	****	0.4536 to 0.7064
+LWL+PA 2 μM vs +LWL+PA 10 μM	0.3031	5.900	Yes	****	0.1767 to 0.4295

Figure S4B

Table Analyzed	Triglyceride
Column A	LWL+ PA
vs	vs
Column B	LWL- PA
Paired t test	
P value	< 0.0001
P value summary	****
Are means signif. different? (P < 0.05)	Yes
One- or two-tailed P value?	Two-tailed
t, df	t=74.86 df=4
Number of pairs	5
How big is the difference?	
Mean of differences	-10.26
95% confidence interval	-10.64 to -9.881
R square	0.9993
How effective was the pairing?	
Correlation coefficient (r)	0.9552
P Value (one tailed)	0.0057
P value summary	**
Was the pairing significantly effective?	Yes

Figure S4C

Table Analyzed	Free fatty acid
Column A	LWL+ PA
vs	vs
Column B	LWL- PA
Paired t test	
P value	< 0.0001
P value summary	****
Are means signif. different? (P < 0.05)	Yes
One- or two-tailed P value?	Two-tailed
t, df	t=362 df=4
Number of pairs	5
How big is the difference?	
Mean of differences	0.89
95% confidence interval	0.88 to 0.89
R square	1.0
How effective was the pairing?	
Correlation coefficient (r)	0.90
P Value (one tailed)	0.0201
P value summary	*
Was the pairing significantly effective?	Yes
